# Rising mortality rates linked to type‐2 diabetes and obesity in the United States: An observational analysis from 1999 to 2022

**DOI:** 10.1111/jdi.14386

**Published:** 2024-12-19

**Authors:** Mushood Ahmed, Abdullah Nofal, Aimen Shafiq, Hira Javaid, Areeba Ahsan, Zain Ali Nadeem, Raheel Ahmed, Mahboob Alam, Mamas A. Mamas, Marat Fudim, Gregg C. Fonarow

**Affiliations:** ^1^ Department of Medicine Rawalpindi Medical University Rawalpindi Pakistan; ^2^ Department of Medicine Services Institute of Medical Sciences Lahore Pakistan; ^3^ Department of Medicine Dow University of Health Sciences Karachi Pakistan; ^4^ Department of Medicine Allama Iqbal Medical College Lahore Pakistan; ^5^ Department of Medicine Foundation University Medical College Islamabad Pakistan; ^6^ National Heart and Lung Institute Imperial College London London UK; ^7^ Department of Cardiology Royal Brompton Hospital London UK; ^8^ Division of Cardiology, The Texas Heart Institute Baylor College of Medicine Houston Texas USA; ^9^ Keele Cardiovascular Research Group, Centre for Prognosis Research Keele University Stoke‐On‐Trent UK; ^10^ Department of Medicine Duke University Medical Center Durham North Carolina USA; ^11^ Duke Clinical Research Institute Durham North Carolina USA; ^12^ Ahmanson‐UCLA Cardiomyopathy Center, Division of Cardiology University of California Los Angeles Los Angeles California USA

**Keywords:** Mortality, Obesity, Type 2 diabetes

## Abstract

**Background:**

The prevalence of type 2 diabetes (T2D) and obesity are increasing in the United States. However, population‐level data for mortality trends due to T2D and obesity are limited. This study aims to assess these death trends among adults in the United States categorized by sex, race, and geographical location.

**Methods:**

We queried the CDC‐WONDER database for multiple cause of death data of adults aged ≥25 years. The crude mortality rates (CMR), age‐adjusted mortality rates (AAMRs), annual percent change (APC), and the average APC (AAPC) along with a 95% confidence interval (CI) were analyzed.

**Results:**

From 1999 to 2022, a total of 88,597 T2DM and obesity‐related deaths were recorded in the United States. The AAMR consistently increased from 1999 to 2017 (APC: 7.64; 95% CI: 1.91–9.96), followed by a marked rise from 2017 to 2022 (APC: 20.13; 95% CI: 12.88–38.57). The AAMR was approximately 3.58 times higher during the COVID‐19 pandemic compared to the period from 1999 to 2019. The AAMR for males was consistently greater than that for females. The highest AAMR was observed in non‐Hispanic (NH) Blacks or African Americans, followed by NH White, Hispanic or Latino, and other NH populations. Rural areas (AAMR: 1.86, 95% CI: 1.83–1.89) exhibited a greater AAMR than urban regions 1.26 (95% CI: 1.25–1.27).

**Conclusions:**

Our results indicate a substantial increasing trend of T2D and obesity‐related deaths in the United States especially during the COVID‐19 pandemic.

## Graphical abstract

This study analyzed mortality trends related to type 2 diabetes and obesity in the United States from 1999 to 2022, revealing a significant increase in AAMRs, particularly during the COVID‐19 pandemic. The highest AAMRs were observed among non‐Hispanic Blacks, males, and individuals in rural areas. The findings highlight a growing public health concern, especially in vulnerable populations.

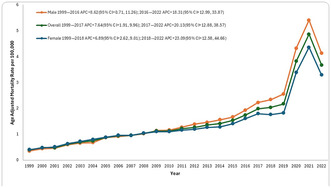

## INTRODUCTION

In the last two decades, obesity has grown to become one of the most widespread noncommunicable diseases (NCD)^1^, ^2^, with the prevalence of obesity almost doubling globally^3^. Over 39% of adults were overweight in 2014, with 13% being obese. Type 2 diabetes (T2D) makes up about 90% of the 537 million cases of diabetes that have been documented globally and by 2045, this figure is predicted to increase to 783 million cases^4^. Obesity and T2D are most closely inter‐related^5^, and by 2025, the number of individuals with diabetes due to obesity is expected to double, reaching 300 million^6^. Currently, over 80% of people with T2D are considered overweight or obese^7^. This can increase the risk of mortality by 7‐folds^8^. According to the World Health Organization (WHO), T2D and obesity are becoming an alarmingly serious global health problem^9^.

The prevalence of T2D and obesity is increasing in the United States^10^. Additionally, it has been demonstrated that one of the key elements for the emergence of diabetes is the obesity pandemic brought on by changes in people's food and lifestyle^11^, which is a major contributing factor to the rise in diabetes prevalence in the United States. The United States has seen significant social and economic growth in the past few decades, which has changed people's lifestyles and increased the incidence of obesity. Other factors contributing to this trend include poor dietary habits, longer sitting times, less exercise, and increased life and work stress^12^. Nevertheless, data regarding mortality trends among individuals with concomitant T2D and obesity is limited. Considering this literature gap, the current study aims to evaluate the mortality burden associated with concomitant type 2 diabetes and obesity in the United States, and to analyze trends in mortality rates over the past two decades. Additionally, the study aims to assess mortality rates stratified by sex, race, and geographical region to identify vulnerable populations. This information is crucial for shaping healthcare policies, offering decision‐makers valuable data on necessary measures to reduce mortality, and helping to assess the effectiveness of previously implemented policies.

## METHODS

### Study setting and population

We analyzed mortality data related to T2DM and obesity obtained from the Centers for Disease Control and Prevention Wide‐Ranging Online Data for Epidemiologic Research (CDC‐WONDER) Database^13^. CDC‐WONDER is a comprehensive database that contains death certificate data from all fifty states and the District of Columbia. We used the Multiple Cause of Death Public Use Record to retrieve data about patients who died with both T2D and obesity as either an underlying cause or contributing cause of death in the United States from 1999 to 2022. We collected death records for patients aged 25 and older using the following International Classification of Diseases, 10th Revision, Clinical Modification codes: E11.0–E11.9 AND E66.0–E66.9. Other researchers have used these same codes to identify T2DM and obesity in administrative databases^14^, ^15^. Additionally, we followed the guidelines established by the reporting standards of the Strengthening the Reporting of Observational Studies in Epidemiology (STROBE)^16^. The study did not require approval from the local institutional review board because it used an anonymous public data set provided by the government.

### Data extraction

The extracted data included population, year, and demographics such as sex, race/ethnicity, age, and regional details. Race/ethnicity was classified into the following categories: Hispanic or Latino, Non‐Hispanic (NH) Black or African American, NH White, and NH other groups (NH Asian or Pacific Islander, Hawaiians, NH American Indian or Alaska Native, etc). The Urban–Rural classification was based on the National Center for Health Statistics Urban–Rural Classification Scheme. The population was divided into urban (large metropolitan area, medium/small metropolitan area) and rural (population < 50,000) counties according to the 2013 U.S. census classification^17^. Regions were classified as Northeast, Midwest, South, and West based on U.S. Census Bureau definitions^18^.

### Statistical analysis

The crude and age‐adjusted mortality rates (CMRs and AAMRs) per 100,000 individuals from 1999 to 2022 were calculated to analyze the trends in mortality related to T2D and obesity. CMRs were calculated by dividing the deaths related to T2D and obesity by the total U.S. population each year. AAMRs were calculated by standardizing T2D and obesity‐related deaths to the 2000 U.S. population, with 95% confidence intervals^19^. The AAMRs were used to analyze mortality patterns across various demographic classifications. The trends in AAMR were determined using the Joinpoint Regression Program (Joinpoint Version 5.1.0, National Cancer Institute) that reported annual percentage change (APC) along with 95% CI^20^. Significant changes in AAMR over time were assessed using log‐linear regression models to examine temporal variations. APCs were considered to increase or decrease if the slope representing the change in mortality significantly deviated from zero, as determined by 2‐tailed *t*‐tests. A sensitivity analysis was performed to identify the death records that listed T2D alone as the primary or contributing cause of death. A *P*‐value of less than 0.05 indicated statistical significance.

## RESULTS

### Annual trends for T2D and obesity‐related AAMR

**Figure 1:**
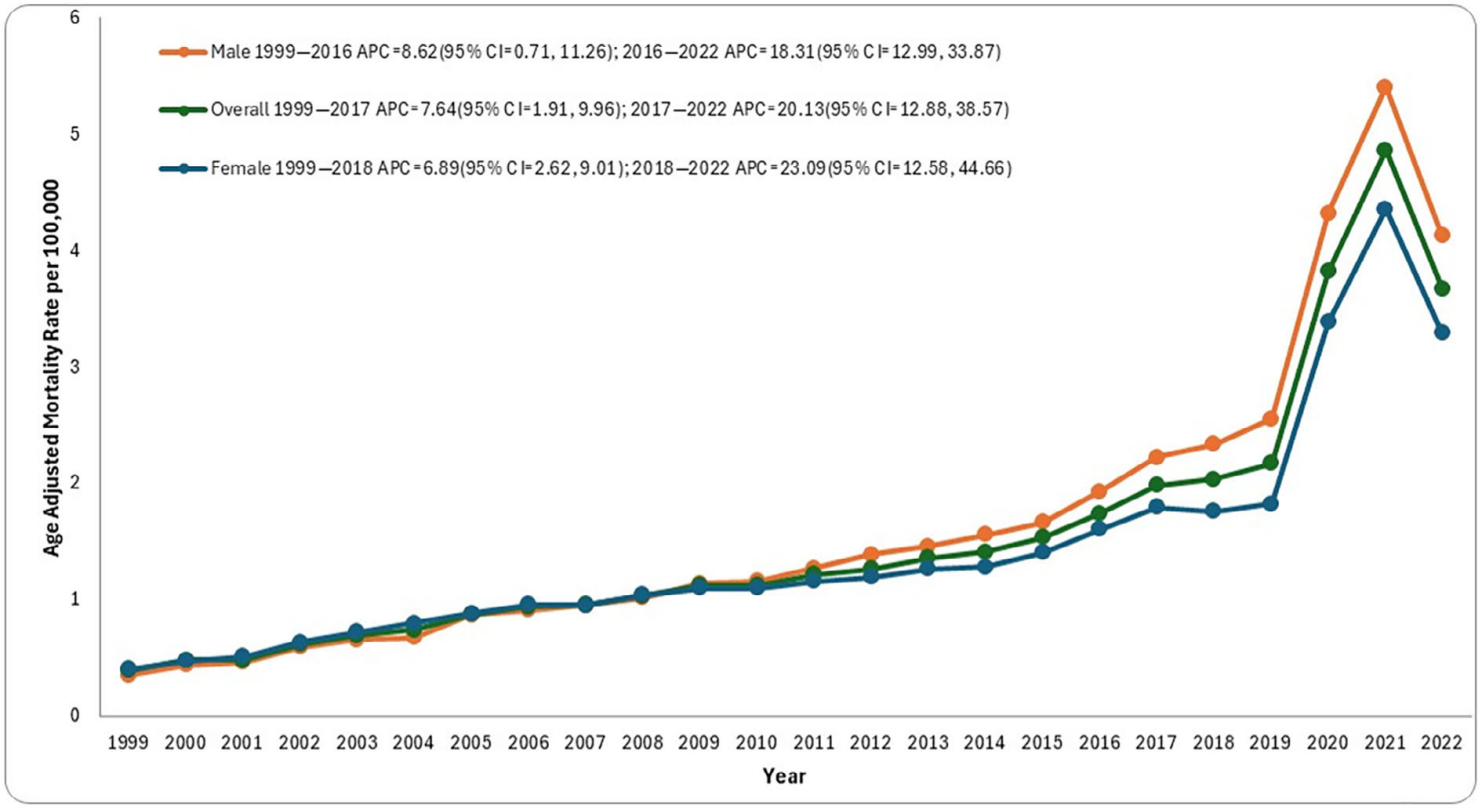
Overall and sex‐stratified type 2 diabetes mellitus and obesity‐related age‐adjusted mortality rates (AAMRs) per 100,000 individuals in the United States, 1999–2022.

**Figure 2:**
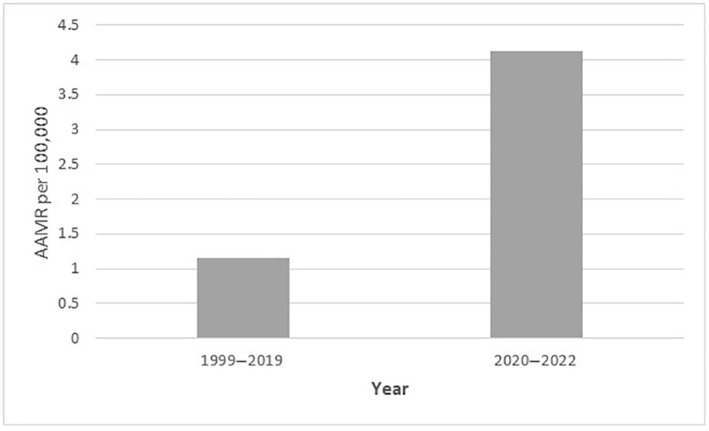
Comparison of age‐adjusted mortality rates (AAMRs) per 100,000 individuals before and during COVID‐19 pandemic.

T2D and obesity led to 88,597 deaths among adults in the United States from 1999 to 2022 (Table S1). The overall AAMR for T2D and obesity‐related deaths increased progressively from 0.39 (95% CI: 0.37–0.40) in 1999 to 3.67 (95% CI: 3.6–3.74) in 2022. The AAMR first increased steadily from 1999 to 2017 (APC: 7.64; 95% CI: 1.91–9.96; *P* < 0.030), then increased steeply till 2022 (APC: 20.13; 95% CI: 12.88–38.57, *P* < 0.000001; Figure 1, Table S2). This increase was primarily observed during the COVID‐19 pandemic, as the AAMR from 2020 to 2022 was nearly 3.58 times higher than that recorded during the rest of the study period (Table S3, Figure 2).

### T2D and obesity‐related AAMR stratified by sex

Males had higher overall AAMR than females in most of the study period. A steady increasing trend was seen in AAMR for males from 1999 to 2016 (APC: 8.62; 95% CI: 0.71–11.26; *P* < 0.045). However, from 2016 to 2022 a sharp rise in mortality rate was observed (APC: 18.31; 95% CI = 12.99–33.87; *P* < 0.000001). A similar rising trend was observed throughout the study period for females. From 1999 to 2018, the mortality rate increased steadily (APC: 6.89; 95% CI = 2.62–9.01, *P* < 0.026). From 2018 to 2022, the mortality rate for females inclined steeply (APC: 23.09; 95% CI: 12.58–44.66; *P* < 0.000001; Figure 1).

### T2D and obesity‐related AAMR stratified by race and ethnicity

Regarding race/ethnicity, the highest AAMRs were observed among NH Black or African American, followed by NH White, Hispanic or Latino, and other NH populations (NH American Indian or Alaska Native and NH Asian or Pacific Islander, Table S4).

AAMR for Black or African Americans in 1999 was 0.55 which gradually increased to 2.2 in 2017 (APC: 6.82; 95% CI: 0.07–9.73; *P* = 0.049). A sharp rise in AAMR was observed from 2017 till the end of the study period (APC: 22.37, 95% CI: 13.40–45.86; *P* < 0.000001).

AAMR for NH whites increased from 1999 to 2017 (APC: 7.68; 95% CI: 4.92–9.34, *P* < 0.01). From 2017 to 2022 a steep increase in mortality rate was observed (APC: 18.29; 95% CI = 13.03–31.30; *P* < 0.000001).

**Figure 3:**
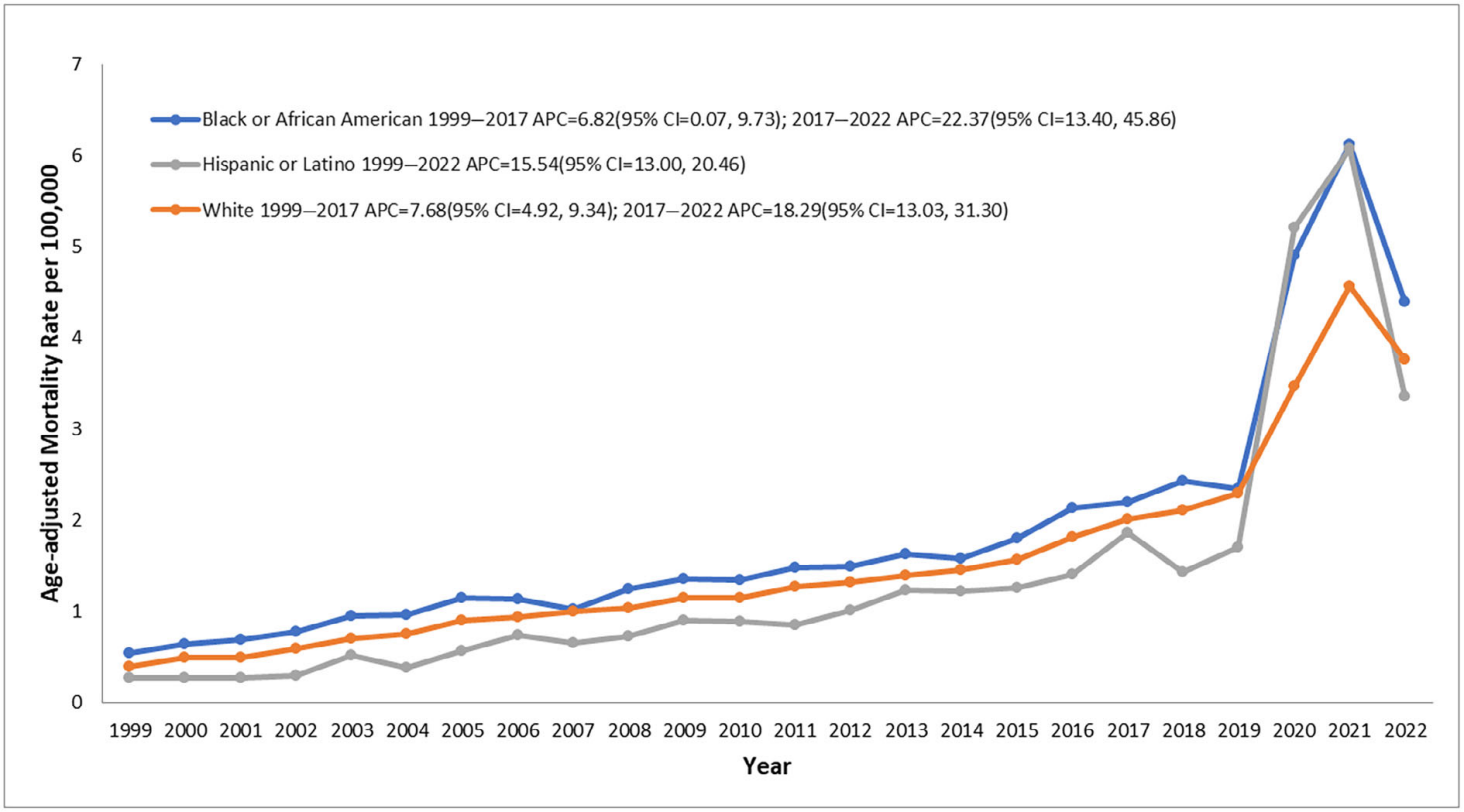
Type 2 diabetes mellitus and obesity‐related age‐adjusted mortality rates (AAMRs) per 100,000 individuals stratified by race and ethnicity in the United States, 1999–2022.

Hispanics or Latinos showed a steady rise in AAMR from 1999 till the end of the study period, that is, 2022 (APC: 15.54; 95% CI: 13.00–20.46; *P* = 0.000001 Figure 3).

Sufficient data were not available due to confidentiality reasons for NH other group to draw a mortality trend.

### T2D and obesity‐related AAMR stratified by geographical region

From 1999 till 2020 variations were observed in mortality rates of different states with the highest AAMR of 3.13 (95% CI: 2.79–3.46) for Vermont followed by an AAMR of 2.67 (95% CI: 2.55–2.80) for Oregon and AAMR of 2.63 (95% CI: 2.52–2.74) for Minnesota and Iowa (95% CI: 2.49–2.77). States with the lowest AAMR were Massachusetts (AAMR: 0.47; 95% CI: 0.44–0.52), Connecticut (AAMR: 0.52; 95% CI: 0.47–0.582), and New York (AAMR: 0.59; 95% CI: 0.57–0.62, Table S5).

In terms of Census Region, the highest AAMR was observed in the West with an AAMR of 6.92 in 2021, followed by the Midwest (highest AAMR 5.65 in 2021), South and the lowest AAMR was observed in the Northeast region (highest AAMR 2.62 in 2021, Figure S1 and Table S6). For the Northeast region the AAMR showed a steady AAMR from 1999 till 2017 (APC: 5.47; 95% CI: 2.12–23.43 *P* = 0.024395) with an increase in AAMR from 2017 till 2020 (APC: 33.51; 95% CI: −4.4 to 42.38 *P* = 0.069586). A steep decline in AAMR was observed from 2020 to 2022 (APC: −1.92; 95% CI: −16.25 to 19.63 *P* = 0.771446).

For the Midwest region the AAMR followed a similar trend, an increase in AAMR from 1999 to 2016 (APC: 7.25; 95% CI: 3.68–9.29 *P* = 0.012797) was observed, with a steep increase in AAMR from 2016 till 2022 (APC: 17.36; 95% CI: 12.93–29.38 *P* < 0.000001).

**Figure 4:**
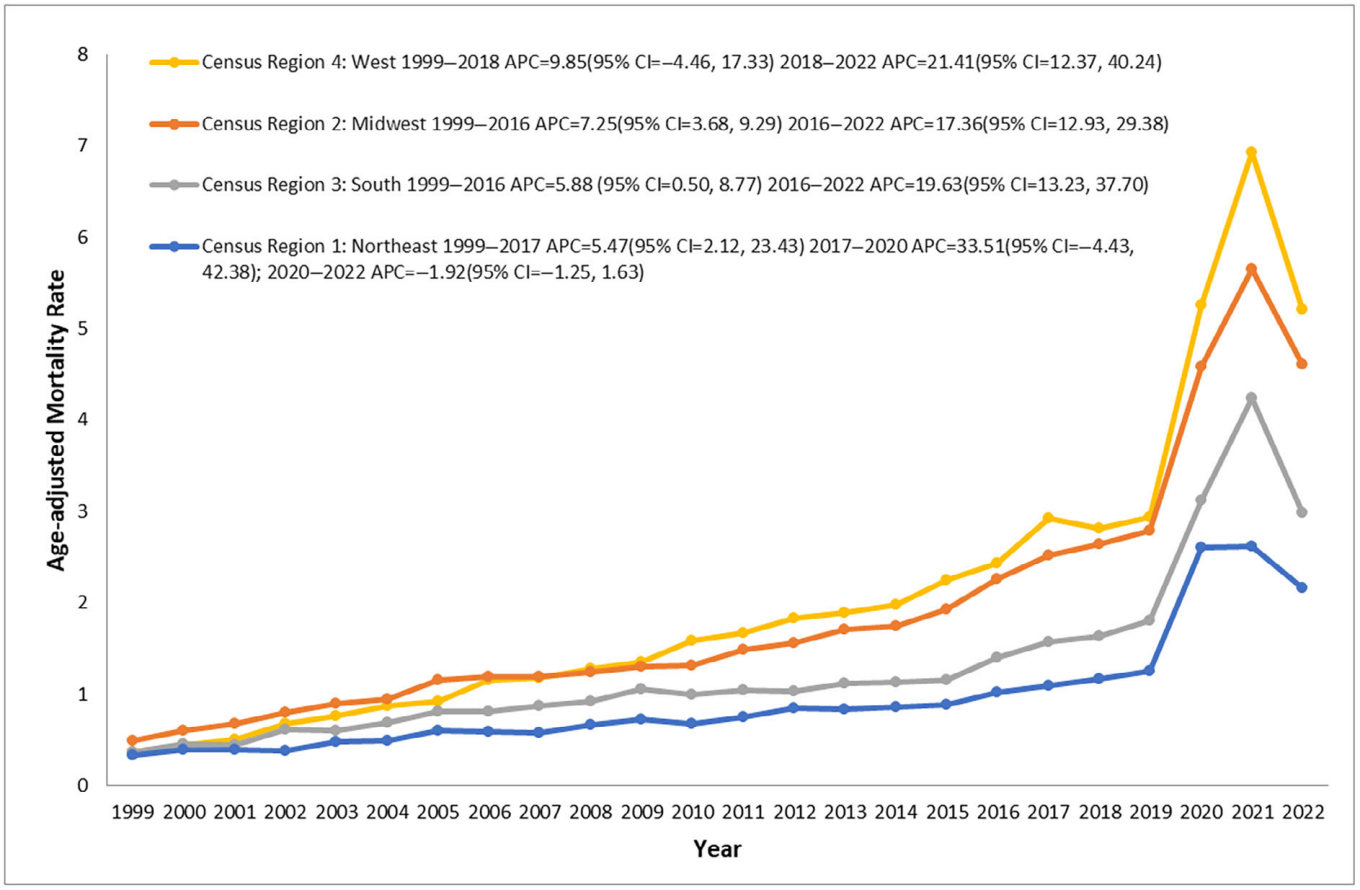
Type 2 diabetes mellitus and obesity‐related age‐adjusted mortality rates (AAMRs) per 100,000 individuals stratified by census Region in the United States, 1999–2022.

Similarly, the Southern region showed a steady increase in AAMR from 1999 till 2016 (APC: 5.88; 95% CI: 0.50–8.77 *P* = 0.040792) and a sharp increase in AAMR from 2016 till 2022 (APC: 19.63; 95% CI: 13.23–37.70 *P* < 0.000001, Figure 4).

For the Western region, AAMR increased steadily from 1999 till 2018 (APC: 9.85; 95% CI: −4.46 to 17.33 *P* 0.066387), and a sharp increase in AAMR from 2018 to 2022 was observed (APC: 21.41; 95% CI: 12.37–40.24 *P* < 0.000001).

### T2D and obesity‐related AAMR stratified by urbanization

**Figure 5:**
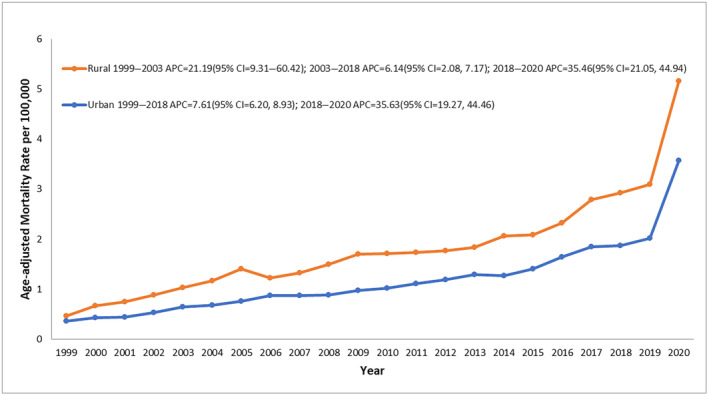
Type 2 diabetes mellitus and obesity‐related age‐adjusted mortality rates (AAMRs) per 100,000 individuals stratified by urbanization in the United States, 1999–2020. AAMRs for urbanizations for 2021 and 2022 are unavailable.

From 1999 to 2020, rural areas consistently exhibited a greater mortality rate than urban areas. Overall AAMR for rural areas was 1.86 (95% CI: 1.83–1.89) and for urban areas, AAMR was 1.26 (95% CI: 1.25–1.27, Table S7). The mortality rate for rural areas increased steadily from 1999 to 2003 (APC: 21.19; 95% CI: 9.31–60.42, *P* < 0.00001) followed by a decline in mortality till 2018 (APC: 6.15; 95% CI: 2.08–7.17; *P* = 0.03, Figure 5). An incline in AAMR was observed from 2018 to 2020 (APC: 35.46; 95% CI: 21.05–44.94; *P* < 0.000001). Urban areas followed a similar trend with steadily increasing AAMR from 1999 to 2018 (APC: 7.61; 95% CI: 6.20–8.93; *P* < 0.000001) with a sharp increase in mortality rate from 2018 to 2020 (APC: 35.64; 95% CI: 19.27–44.46, *P* < 0.000001). Data for AAMR was unavailable for 2021 and 2022 in the CDC Wonder Database.

### Sensitivity analysis

The sensitivity analysis identified 1,883,120 records that listed T2D as the primary or contributing cause of death from 1999 to 2022 (Table S8). T2D‐related mortality also demonstrated an increasing trend over the study period.

## DISCUSSION

Our CDC WONDER analysis provides insights into the disparities in T2D and obesity‐related mortality in the United States over two decades. These findings underscore the complexity of T2D and its association with obesity, highlighting critical areas for targeted intervention and policy development. The substantial variations in the mortality levels across sex, race, age, and geographical location, signal the inequities related to diabetes care in America, affecting all levels of the health system^12^, ^21^.

Between 1999 and 2017, the AAMR for T2D and obesity‐related mortality increased steadily, then inclined steeply till 2022. The initial pattern points to a worsening of the obesity‐related T2D epidemic^22^, most likely because of growing adult obesity rates, which reached 41.9% in 2020^23^. Whereas the sharp increase till 2022 points toward the COVID‐19 pandemic. The mortality rates almost tripled during the COVID‐19 pandemic from 2020 to 2022 when compared with the rest of the study period. The COVID‐19 pandemic has had a profound influence on the AAMR for T2D and obesity‐related fatalities. People with underlying medical issues, especially those with T2D and obesity, were disproportionately impacted by COVID‐19 and were more likely to have severe illness or die. The pandemic worsened already‐existing health inequities in addition to placing a burden on healthcare systems and perhaps causing chronic condition care to be delayed or insufficient.

This study revealed two phases in AAMR trends: a gradual increase from 1999 to 2017, followed by a sharp rise after 2017. Initially, cardiovascular disease or malignancy likely contributed to the high AAMR, but after 2017, especially during the COVID‐19 pandemic, the causes may have shifted. These trends in patients with T2D and obesity may explain these changes.

Moreover, the sharp rise in the AAMR for T2D and obesity‐related deaths from 2017 to 2022 is a reflection of how the COVID‐19 pandemic worsened existing health disparities. Communities that were already facing high rates of T2D and obesity—like Black Americans, low‐income families, and those living in rural areas—were hit especially hard. These groups often struggle with limited access to healthcare and are more likely to have underlying health conditions. When COVID‐19 struck, it magnified these vulnerabilities, leading to a significant increase in deaths. The steady rise in mortality from 1999 to 2017 was troubling, but the steep spike after 2020 shows how devastating the pandemic was, particularly for those who were already at risk. COVID‐19 did not just affect everyone equally—it disproportionately harmed those who were already most vulnerable, revealing deep inequities in our healthcare system.

According to our analysis, the AAMR of males was marginally greater than that of females, which is consistent with previous research showing that males with T2D have a higher mortality risk because of variables like worse glycemic control, a higher frequency of CV problems, and possibly a delay in seeking medical attention as compared to women^24^. Moreover, unhealthy habits like drinking and smoking may be made worse by increased social and competitive pressures, which raises the risk of diabetes‐related mortality in men^25^.

Our analysis reveals noticeable differences in T2D and obesity‐related mortality between different racial and ethnic groupings. The greatest AAMR is seen among NH Black or African Americans. A complicated interaction between genetic predispositions, socioeconomic circumstances, healthcare access, structural racism and cultural variations in nutrition and lifestyle can be credited to these inequities^26^. For example, NH Black or African Americans have high poverty rates and restricted access to healthcare, which can raise their mortality rates. Additionally, long‐term treatment of diabetes is more costly than other chronic illnesses, which puts a significant financial strain on both the present healthcare system and each family's overall well‐being.

Our analysis shows significant regional variance in AAMRs, with the West showing the highest rates, followed by the Midwest, South, and Northeast. States with especially high AAMRs are Vermont and Oregon. Furthermore, compared to the urban areas, death rates are greater in rural locations. These trends can result from regional variations in the healthcare system, the accessibility of medical services, socioeconomic position, and lifestyle choices. Higher death rates are a result of rural communities' frequently restricted access to preventative treatment and healthcare services. Even though America's rural areas have developed medical services and insurance, their capacity remains significantly lower than that of urban areas, which results in inadequate diabetes care and management^27^. Additionally, socioeconomic status is also an important factor that can affect the prevalence of diabetes and its complications. As a result of factors influencing access and availability of care, up to 70% of diabetes cases go undetected globally. This number reaches 39% in the United States^28^. A previous study which aimed to assess cardiovascular diseases and type‐2 diabetes‐related mortality burden also identified significant urban–rural and demographic disparities. While analyzing this high proportion, many factors must be considered; inadequate access to high‐quality healthcare might prove crucial.

Since mortality is sensitive to reflect health systems' efficiency^21^, it is commonly used to evaluate the quality of medical care. And in this study, we found increasing mortality rates for T2D and obesity in the United States revealing disparities across sociodemographic groups. Considering this, a comprehensive strategy that considers comorbidities and combines efforts to guarantee that patients have access to comprehensive, high‐quality medical care at every level of the health system is needed to reduce diabetes and obesity‐related mortality^21^. In this strategy, primary care institutions are crucial. Standardized guidelines for the care and monitoring of diabetes, its associated risk factors, and other NCDs are provided by the WHO^29^, ^30^. These are important tools that, if properly put into practice, can assist the United States in raising the standard of diabetes and obesity treatment for its citizens.

Some limitations should be considered while interpreting our findings. The research relied on the data obtained from death certificates and co‐existing pathologies such as cardiovascular diseases may have influenced our findings. Moreover, the findings of sensitivity analysis are striking with 1,883,120 deaths being attributed to T2D. Studies have estimated that around 80–90% of individuals with T2D are overweight or obese^31^, ^32^. However, CDC WONDER listed less than 5% of deaths attributable to concomitant T2D and obesity. Although it is plausible that the 1,883,120 individuals who died from T2D had a much higher prevalence of co‐existing obesity, it was not mentioned on patients' death certificates. This is one of the major limitations of the CDC‐WONDER database because if the treating provider who is signing the death certificate does not also include key comorbidities as contributing factors (i.e., obesity) they go unrecognized as contributing to the mortality event. Moreover, the CDC WONDER database does not include information on trends in life expectancy and prevalence of T2D and obesity in the United States. This highlights the need for better reporting of comorbidities like obesity on death records.

Our findings indicate a concerning increase in T2D and obesity‐related fatalities in the United States, especially during the COVID‐19 pandemic. Men, NH Black or African American, and residents of the West and rural regions have the greatest mortality rates. The main goals of public health initiatives should be to ensure that these communities have improved access to healthcare facilities, more knowledge about T2D and obesity prevention, and assistance in establishing healthier surroundings. Reducing socioeconomic inequities and enhancing access to care are two underlying concerns that need to be addressed if we are to significantly cut the death rates of individuals most impacted by obesity and T2D.

## FUNDING INFORMATION

No financial support was received for the study.

## DISCLOSURE

Dr Fonarow reported receiving personal fees from Abbott, Amgen, AstraZeneca, Bayer, Boehringer Ingelheim, Cytokinetics, Eli Lilly, Johnson & Johnson, Medtronic, Merck, Novartis, and Pfizer outside the submitted work. Dr Fudim reported receiving personal fees from Alleviant, Ajax, Alio Health, Alleviant, Artha, Audicor, Axon Therapies, Bayer, Bodyguide, Bodyport, Boston Scientific, Broadview, Cadence, Cardioflow, Cardionomics, Coridea, CVRx, Daxor, Deerfield Catalyst, Edwards LifeSciences, Echosens, EKO, Feldschuh Foundation, Fire1, FutureCardia, Galvani, Gradient, Hatteras, HemodynamiQ, Impulse Dynamics, Intershunt, Medtronic, Merck, NIMedical, NovoNordisk, NucleusRx, NXT Biomedical, Orchestra, Pharmacosmos, PreHealth, Presidio, Procyreon, ReCor, Rockley, SCPharma, Shifamed, Splendo, Summacor, SyMap, Verily, Vironix, Viscardia, and Zoll; and receiving grants from the National Institutes of Health, Doris Duke, outside the submitted work. No other disclosures were reported.

Approval of the research protocol: N/A.

Informed consent: N/A.

Approval date of registry and the registration no. of the study/trial: N/A.

Animal studies: N/A.

## Supporting information


**Table S1.** T2DM and obesity related deaths, stratified by Sex and Race in the United States, 1999–2022.
**Table S2.** Annual percent change (APC) of T2DM related age‐adjusted mortality rates per 100,000 in the United States, 1999–2022.
**Table S3.** Overall and sex‐stratified type 2 diabetes mellitus and obesity related age‐adjusted mortality rates per 100,000 in the United States, 1999–2022.
**Table S4.** Type 2 diabetes mellitus and obesity related age‐adjusted mortality rates per 100,000, stratified by race in the United States, 1999–2022.
**Table S5.** Type 2 diabetes mellitus and obesity related age‐adjusted mortality rates per 100,000, stratified by States in the United States, 1999–2022.
**Table S6.** Type 2 diabetes mellitus and obesity related age‐adjusted mortality rates per 100,000, stratified by census region in the United States, 1999–2022.
**Table S7.** Type 2 diabetes mellitus and obesity related age‐adjusted mortality rates per 100,000 in United States stratified by Urban‐Rural Classification, 1999–2020.
**Table S8.** Deaths due to T2D as primary or contributing cause of death.
**Figure S1.** Type 2 Diabetes Mellitus and Obesity‐Related Annual Percentage Change (APC) in the United States from 1999 to 2022 stratified by (A) Region (B) Sex (C) Race and (D) Urbanization.

## Data Availability

All data generated or analyzed during this study are included in this article. Further inquiries can be directed to the corresponding author.
