# Supplementary material for: Rising mortality rates linked to type‐2 diabetes and obesity in the United States: An observational analysis from 1999 to 2022
Source: J Diabetes Investig. 2024 Dec 19;16(3):492–500. doi: 10.1111/jdi.14386 (PMC11871392; doi:10.1111/jdi.14386)
Supplement: Supplementary file 1 — Table S1. T2DM and obesity related deaths, stratified by Sex and Race in the United States, 1999–2022. Table S2. Annual percent change (APC) of T2DM related age‐adjusted mortality rates per 100,000 in the United States, 1999–2022. Table S3. Overall and sex‐stratified type 2 diabetes mellitus and obesity related age‐adjusted mortality rates per 100,000 in the United States, 1999–2022. Table S4. Type 2 diabetes mellitus and obesity related age‐adjusted mortality rates per 100,000, stratified by race in the United States, 1999–2022. Table S5. Type 2 diabetes mellitus and obesity related age‐adjusted mortality rates per 100,000, stratified by States in the United States, 1999–2022. Table S6. Type 2 diabetes mellitus and obesity related age‐adjusted mortality rates per 100,000, stratified by census region in the United States, 1999–2022. Table S7. Type 2 diabetes mellitus and obesity related age‐adjusted mortality rates per 100,000 in United States stratified by Urban‐Rural Classification, 1999–2020. Table S8. Deaths due to T2D as primary or contributing cause of death. Figure S1. Type 2 Diabetes Mellitus and Obesity‐Related Annual Percentage Change (APC) in the United States from 1999 to 2022 stratified by (A) Region (B) Sex (C) Race and (D) Urbanization. [file JDI-16-492-s001.docx]

**Supplementary Appendix**

**Supplemental Table 1: T2DM and obesity related deaths, Stratified by Sex and Race in the United States, 1999 to 2022**

|  | Deaths | | | | | |
| --- | --- | --- | --- | --- | --- | --- |
| Year | **Female** | **Male** | **NH White** | **NH Black or African American** | **NH others** | **Hispanic or Latino** |
| 1999 | 388 | 289 | 546 | 94 | Suppressed | 29 |
| 2000 | 477 | 371 | 689 | 110 | 13 | 31 |
| 2001 | 541 | 397 | 772 | 123 | Suppressed | 33 |
| 2002 | 639 | 497 | 926 | 145 | 22 | 42 |
| 2003 | 739 | 579 | 1043 | 176 | 23 | 72 |
| 2004 | 837 | 621 | 1189 | 175 | 31 | 57 |
| 2005 | 969 | 801 | 1435 | 220 | 30 | 84 |
| 2006 | 1031 | 851 | 1502 | 229 | 37 | 109 |
| 2007 | 1079 | 913 | 1628 | 208 | 36 | 118 |
| 2008 | 1152 | 990 | 1684 | 267 | 53 | 133 |
| 2009 | 1280 | 1139 | 1898 | 296 | 49 | 167 |
| 2010 | 1284 | 1194 | 1936 | 298 | 68 | 172 |
| 2011 | 1414 | 1300 | 2143 | 334 | 68 | 165 |
| 2012 | 1483 | 1491 | 2335 | 364 | 63 | 207 |
| 2013 | 1588 | 1611 | 2474 | 385 | 74 | 260 |
| 2014 | 1643 | 1731 | 2637 | 375 | 76 | 283 |
| 2015 | 1848 | 1908 | 2868 | 459 | 106 | 310 |
| 2016 | 2117 | 2239 | 3322 | 558 | 109 | 356 |
| 2017 | 2440 | 2645 | 3821 | 600 | 145 | 508 |
| 2018 | 2435 | 2850 | 4056 | 674 | 136 | 406 |
| 2019 | 2620 | 3121 | 4385 | 657 | 176 | 514 |
| 2020 | 4809 | 5353 | 6751 | 1400 | 389 | 1602 |
| 2021 | 6094 | 6769 | 8653 | 1731 | 511 | 1944 |
| 2022 | 4744 | 5286 | 7298 | 1262 | 352 | 1092 |

NH, non-Hispanic.

Suppressed: Rates are suppressed for statistics representing zero to nine (0-9) deaths in years 1999 and later.

(This is done to protect the personal privacy of individuals)

**Supplemental Table 2: Annual percent change (APC) of T2DM Related Age-Adjusted Mortality Rates per 100,000 in the United States, 1999 to 2022**

| **Year Interval** | **APC (95% CI)** |
| --- | --- |
| **Overall** |  |
| 1999-2017 | 7.6415 (1.9155 - 9.9676) |
| 2017-2022 | 20.1353 (12.8869 - 38.5742) |
| **Male** |  |
| 1999-2016 | 8.6201 (0.7183 - 11.2649) |
| 2016-2022 | 18.3196 (12.9954 - 33.8732) |
| **Female** |  |
| 1999-2018 | 6.8981 (2.6286 - 9.0156) |
| 2018-2022 | 23.0907 (12.5893 - 44.666) |
| **Race** |  |
| **NH White** |  |
| 1999-2017 | 7.6842 (4.9261 - 9.3427) |
| 2017-2022 | 18.2964 (13.0381 - 31.3005) |
| **NH Black or African American** |  |
| 1999-2017 | 6.8213 (0.0715 - 9.7396) |
| 2017-2022 | 22.3764 (13.4023 - 45.869) |
| **Hispanic or Latino** |  |
| 1999-2022 | 15.5499 (13.0053 - 20.4623) |
| **Urbanization** |  |
| **Rural** |  |
| 1999-2003 | 21.19 (9.32-60.43) |
| 2003-2018 | 6.15 (2.08-7.18) |
| 2018-2020 | 35.47 (21.05-44.94) |
| **Urban** |  |
| 1999-2018 | 7.61 (6.21-8.93) |
| 2018-2020 | 35.64 (19.28-44.47) |
| **Census Region** |  |
| **Northeast** |  |
| 1999-2017 | 5.4781 (2.124 - 23.4395) |
| 2017-2020 | 33.5193 (-4.4356 - 42.3899) |
| 2020-2022 | -1.9234 (-16.2582 - 19.6391) |
| **Midwest** |  |
| 1999-2016 | 7.2576 (3.6846 - 9.2992) |
| 2016-2022 | 17.3692 (12.9318 - 29.3895) |
| **South** |  |
| 1999-2016 | 5.8854 (0.5052 - 8.7782) |
| 2016-2022 | 19.6388 (13.2318 - 37.7055) |
| **West** |  |
| 1999-2018 | 9.8536 (-4.4679 - 17.3391) |
| 2018-2022 | 21.4135 (12.3752 - 40.2447) |

**Supplemental Table 3: Overall and Sex‐Stratified** **Type 2 Diabetes mellitus and Obesity Related Age-Adjusted Mortality Rates per 100,000 in the United States, 1999 to 2022**

|  | **Age-Adjusted Rate (95% CI)** | | |
| --- | --- | --- | --- |
| **Year** | **Male** | **Female** | **Overall** |
| **1999** | 0.355 (0.313-0.397) | 0.406 (0.365-0.447) | 0.393 (0.363 - 0.423) |
| **2000** | 0.443 (0.397-0.488) | 0.475 (0.432-0.518) | 0.483 (0.45 - 0.516) |
| **2001** | 0.464 (0.418-0.511) | 0.513 (0.47-0.557) | 0.486 (0.454 - 0.517) |
| **2002** | 0.594 (0.541-0.647) | 0.636 (0.586-0.686) | 0.617 (0.581 - 0.653) |
| **2003** | 0.659 (0.605-0.713) | 0.726 (0.673-0.779) | 0.695 (0.657 - 0.733) |
| **2004** | 0.677 (0.623-0.732) | 0.802 (0.747-0.856) | 0.741 (0.702 - 0.779) |
| **2005** | 0.871 (0.81-0.933) | 0.881 (0.825-0.937) | 0.878 (0.837 - 0.919) |
| **2006** | 0.911 (0.849-0.973) | 0.963 (0.903-1.023) | 0.94 (0.897 - 0.983) |
| **2007** | 0.952 (0.889-1.015) | 0.957 (0.899-1.015) | 0.959 (0.917 - 1.002) |
| **2008** | 1.021 (0.956-1.085) | 1.04 (0.979-1.101) | 1.036 (0.991 - 1.08) |
| **2009** | 1.142 (1.075-1.21) | 1.1 (1.038-1.161) | 1.123 (1.077 - 1.168) |
| **2010** | 1.163 (1.095-1.23) | 1.104 (1.043-1.166) | 1.128 (1.083 - 1.173) |
| **2011** | 1.272 (1.201-1.343) | 1.158 (1.097-1.219) | 1.219 (1.172 - 1.265) |
| **2012** | 1.391 (1.319-1.463) | 1.195 (1.133-1.257) | 1.268 (1.221 - 1.314) |
| **2013** | 1.461 (1.389-1.534) | 1.265 (1.202-1.328) | 1.358 (1.31 - 1.405) |
| **2014** | 1.562 (1.486-1.637) | 1.286 (1.223-1.35) | 1.411 (1.362 - 1.459) |
| **2015** | 1.669 (1.592-1.745) | 1.406 (1.34-1.471) | 1.538 (1.487 - 1.588) |
| **2016** | 1.932 (1.85-2.014) | 1.608 (1.537-1.678) | 1.745 (1.692 - 1.798) |
| **2017** | 2.223 (2.137-2.31) | 1.795 (1.722-1.868) | 1.987 (1.931 - 2.043) |
| **2018** | 2.337 (2.249-2.424) | 1.76 (1.688-1.832) | 2.038 (1.982 - 2.095) |
| **2019** | 2.555 (2.463-2.647) | 1.826 (1.754-1.898) | 2.17 (2.113 - 2.227) |
| **2020** | 4.321 (4.202-4.44) | 3.388 (3.29-3.486) | 3.82 (3.744 - 3.896) |
| **2021** | 5.404 (5.271-5.536) | 4.354 (4.241-4.467) | 4.865 (4.778 - 4.951) |
| **2022** | 4.128 (4.014-4.242) | 3.294 (3.197-3.391) | 3.673 (3.6 - 3.747) |

**Supplemental Table 4: Type 2 Diabetes mellitus and Obesity Related Age-Adjusted Mortality Rates per 100,000, Stratified by Race in the United States, 1999 to 2022**

|  | Age-Adjusted Rate (95% CI) | | | |
| --- | --- | --- | --- | --- |
| Year | **NH White** | **NH Black or African American** | **NH others** | **Hispanic or Latino** |
| 1999 | 0.393  (0.36-0.427) | 0.55  (0.442-0.677) | Suppressed (Suppressed-Suppressed) | 0.275  (0.179-0.403) |
| 2000 | 0.493  (0.456-0.53) | 0.645  (0.523-0.767) | Unreliable (0.091 - 0.325) | 0.28  (0.183-0.411) |
| 2001 | 0.498  (0.463-0.534) | 0.692  (0.567-0.817) | Suppressed (Suppressed - Suppressed) | 0.279  (0.188-0.399) |
| 2002 | 0.599  (0.56-0.638) | 0.775  (0.646-0.903) | 0.288  (0.173 - 0.45) | 0.302  (0.212-0.419) |
| 2003 | 0.709  (0.665-0.752) | 0.959  (0.815-1.103) | 0.275  (0.17 - 0.421) | 0.523  (0.404-0.667) |
| 2004 | 0.753  (0.71-0.796) | 0.971  (0.825-1.117) | 0.364  (0.246 - 0.52) | 0.383  (0.284-0.504) |
| 2005 | 0.904  (0.857-0.951) | 1.152  (0.996-1.307) | 0.33  (0.217 - 0.48) | 0.57  (0.448-0.714) |
| 2006 | 0.943  (0.894-0.991) | 1.141  (0.99-1.292) | 0.38  (0.263 - 0.531) | 0.738  (0.593-0.882) |
| 2007 | 0.998  (0.949-1.046) | 1.033  (0.889-1.176) | 0.361  (0.25 - 0.504) | 0.658  (0.529-0.786) |
| 2008 | 1.042  (0.991-1.093) | 1.244  (1.091-1.398) | 0.542  (0.401 - 0.717) | 0.732  (0.601-0.864) |
| 2009 | 1.149  (1.096-1.202) | 1.364  (1.205-1.524) | 0.466  (0.34 - 0.624) | 0.899  (0.756-1.042) |
| 2010 | 1.15  (1.098-.1202) | 1.353  (1.195-1.511) | 0.598  (0.461 - 0.762) | 0.893  (0.753-1.032) |
| 2011 | 1.272  (1.217-1.327) | 1.482  (1.319-1.646) | 0.529  (0.408 - 0.674) | 0.856  (0.719-0.993) |
| 2012 | 1.327  (1.272-1.382) | 1.501  (1.341-1.66) | 0.535  (0.408 - 0.688) | 1.015  (0.87-1.161) |
| 2013 | 1.399  (1.342-1.455) | 1.631  (1.464-1.798) | 0.583  (0.455 - 0.736) | 1.238  (1.08-1.395) |
| 2014 | 1.462  (1.405-1.519) | 1.58  (1.416-1.744) | 0.572  (0.446 - 0.723) | 1.22  (1.071-1.37) |
| 2015 | 1.566  (1.507-1.625) | 1.804  (1.633-1.974) | 0.742  (0.598 - 0.887) | 1.261  (1.114-1.408) |
| 2016 | 1.817  (1.753-1.881) | 2.141  (1.958-2.324) | 0.748  (0.605 - 0.892) | 1.409  (1.257-1.562) |
| 2017 | 2.014  (1.948-2.079) | 2.2  (2.019-2.381) | 0.955  (0.797 - 1.113) | 1.87  (1.701-2.039) |
| 2018 | 2.118  (2.051-2.186) | 2.434  (2.246-2.622) | 0.865  (0.718 - 1.012) | 1.436  (1.291-1.581) |
| 2019 | 2.298  (2.227-2.369) | 2.352  (2.168-2.537) | 1.1  (0.935 - 1.264) | 1.71 (1.557-1.864) |
| 2020 | 3.464  (3.378-3.55) | 4.899  (4.636-5.163) | 2.297  (2.066 - 2.528) | 5.213  (4.949-5.476) |
| 2021 | 4.566  (4.466-4.667) | 6.124  (5.827-6.421) | 2.675  (2.44 - 2.91) | 6.065  (5.786 - 6.344) |
| 2022 | 3.762  (3.672-3.852) | 4.399  (4.149-4.648) | 1.775  (1.588 - 1.963) | 3.353  (3.147 - 3.559) |

NH = non-Hispanic.

Unreliable: Rates are marked as "unreliable" when the death count is less than 20.

Suppressed: Rates are suppressed for statistics representing zero to nine (0-9) deaths in years 1999 and later.

(This is done to protect the personal privacy of individuals)

**Supplemental Table 5: Type 2 Diabetes mellitus and Obesity Related Age-Adjusted Mortality Rates per 100,000, Stratified by States in the United States, 1999 to 2022**

| **State** | **Age-Adjusted Rate (95%CI)** | **Age-Adjusted Rate (95%CI)** |
| --- | --- | --- |
|  | **1999-2020** | **2021-2022** |
| Alabama | 0.944 (0.875 - 1.014) | 4.881 (4.384 - 5.379) |
| Alaska | 2.454 (2.091 - 2.817) | 4.371 (3.137 - 5.93) |
| Arizona | 1.211 (1.142 - 1.28) | 4.157 (3.781 - 4.532) |
| Arkansas | 0.947 (0.856 - 1.037) | 3.384 (2.847 - 3.921) |
| California | 1.899 (1.863 - 1.936) | 6.436 (6.229 - 6.644) |
| Colorado | 1.894 (1.792 - 1.997) | 6.071 (5.547 - 6.596) |
| Connecticut | 0.524 (0.466 - 0.582) | 1.374 (1.086 - 1.715) |
| Delaware | 1.389 (1.198 - 1.58) | 1.729 (1.175 - 2.454) |
| District of Columbia | 0.576 (0.423 - 0.766) | Unreliable (1.016 - 2.995) |
| Florida | 0.961 (0.928 - 0.994) | 3.115 (2.935 - 3.296) |
| Georgia | 0.539 (0.499 - 0.58) | 1.268 (1.091 - 1.445) |
| Hawaii | 1.047 (0.911 - 1.183) | 2.426 (1.818 - 3.174) |
| Idaho | 2.132 (1.942 - 2.323) | 8.438 (7.376 - 9.499) |
| Illinois | 0.904 (0.862 - 0.947) | 2.525 (2.303 - 2.747) |
| Indiana | 1.839 (1.755 - 1.923) | 4.597 (4.179 - 5.015) |
| Iowa | 2.631 (2.489 - 2.773) | 7.841 (7.064 - 8.619) |
| Kansas | 1.894 (1.765 - 2.024) | 6.408 (5.654 - 7.161) |
| Kentucky | 1.534 (1.441 - 1.627) | 5.144 (4.608 - 5.679) |
| Louisiana | 0.631 (0.571 - 0.691) | 2.007 (1.671 - 2.343) |
| Maine | 1.737 (1.569 - 1.905) | 4.177 (3.389 - 4.965) |
| Maryland | 0.99 (0.924 - 1.056) | 3.569 (3.19 - 3.948) |
| Massachusetts | 0.479 (0.438 - 0.521) | 1.632 (1.395 - 1.868) |
| Michigan | 1.152 (1.099 - 1.205) | 3.775 (3.469 - 4.081) |
| Minnesota | 2.632 (2.521 - 2.743) | 8.924 (8.301 - 9.547) |
| Mississippi | 0.743 (0.662 - 0.823) | 2.382 (1.923 - 2.841) |
| Missouri | 1.269 (1.197 - 1.342) | 3.425 (3.053 - 3.796) |
| Montana | 2.224 (1.998 - 2.449) | 6.668 (5.508 - 7.828) |
| Nebraska | 2.06 (1.893 - 2.227) | 5.724 (4.845 - 6.602) |
| Nevada | 0.649 (0.57 - 0.729) | 2.291 (1.865 - 2.717) |
| New Hampshire | 1.281 (1.131 - 1.43) | 2.871 (2.258 - 3.598) |
| New Jersey | 0.64 (0.599 - 0.681) | 1.416 (1.225 - 1.607) |
| New Mexico | 1.4 (1.268 - 1.531) | 4.778 (4.003 - 5.553) |
| New York | 0.595 (0.568 - 0.622) | 1.773 (1.628 - 1.918) |
| North Carolina | 1.455 (1.393 - 1.516) | 4.158 (3.844 - 4.471) |
| North Dakota | 2.271 (1.99 - 2.552) | 6.226 (4.863 - 7.853) |
| Ohio | 1.824 (1.763 - 1.885) | 4.583 (4.272 - 4.894) |
| Oklahoma | 1.934 (1.822 - 2.046) | 11.243 (10.378 - 12.108) |
| Oregon | 2.675 (2.547 - 2.802) | 7.863 (7.209 - 8.517) |
| Pennsylvania | 1.294 (1.246 - 1.343) | 3.985 (3.717 - 4.253) |
| Rhode Island | 0.723 (0.598 - 0.847) | 2.237 (1.605 - 3.035) |
| South Carolina | 1.209 (1.129 - 1.288) | 2.697 (2.349 - 3.044) |
| South Dakota | 1.815 (1.586 - 2.044) | 7.186 (5.747 - 8.625) |
| Tennessee | 1.679 (1.599 - 1.759) | 4.527 (4.125 - 4.93) |
| Texas | 1.32 (1.28 - 1.359) | 3.871 (3.678 - 4.064) |
| Utah | 1.156 (1.035 - 1.278) | 4.832 (4.148 - 5.515) |
| Vermont | 3.13 (2.798 - 3.463) | 7.967 (6.363 - 9.57) |
| Virginia | 0.903 (0.849 - 0.956) | 2.715 (2.437 - 2.993) |
| Washington | 2.255 (2.163 - 2.347) | 6.659 (6.194 - 7.123) |
| West Virginia | 2.246 (2.079 - 2.412) | 4.943 (4.122 - 5.764) |
| Wisconsin | 2.401 (2.3 - 2.502) | 10.005 (9.363 - 10.647) |
| Wyoming | 2.638 (2.289 - 2.986) | 16.405 (13.742 - 19.068) |

**Supplemental Table 6: Type 2 Diabetes mellitus and Obesity Related Age-Adjusted Mortality Rates per 100,000, Stratified by Census Region in the United States, 1999 to 2022**

| **Census Region** | **Year** | **Age-Adjusted Rate (95% CI)** |
| --- | --- | --- |
| **Northeast** |  |  |
| Northeast | 1999 | 0.332 (0.271-0.393) |
| Northeast | 2000 | 0.395 (0.33-0.46) |
| Northeast | 2001 | 0.391 (0.327-0.454) |
| Northeast | 2002 | 0.383 (0.32-0.446) |
| Northeast | 2003 | 0.485 (0.415-0.556) |
| Northeast | 2004 | 0.495 (0.422-0.567) |
| Northeast | 2005 | 0.598 (0.522-0.675) |
| Northeast | 2006 | 0.589 (0.511-0.666) |
| Northeast | 2007 | 0.572 (0.498-0.646) |
| Northeast | 2008 | 0.663 (0.583-0.743) |
| Northeast | 2009 | 0.728 (0.644-0.812) |
| Northeast | 2010 | 0.681 (0.601-0.761) |
| Northeast | 2011 | 0.744 (0.658-0.83) |
| Northeast | 2012 | 0.848 (0.761-0.934) |
| Northeast | 2013 | 0.833 (0.746-0.92) |
| Northeast | 2014 | 0.855 (0.767-0.942) |
| Northeast | 2015 | 0.887 (0.799-0.976) |
| Northeast | 2016 | 1.022 (0.928-1.117) |
| Northeast | 2017 | 1.096 (0.998-1.194) |
| Northeast | 2018 | 1.17 (1.071-1.268) |
| Northeast | 2019 | 1.257 (1.156-1.359) |
| Northeast | 2020 | 2.608 (2.458-2.757) |
| Northeast | 2021 | 2.622 (2.474 - 2.77) |
| Northeast | 2022 | 2.161 (2.028 - 2.295) |
| **Midwest** |  |  |
| Midwest | 1999 | 0.487 (0.419-0.555) |
| Midwest | 2000 | 0.597 (0.523-0.671) |
| Midwest | 2001 | 0.675 (0.597-0.754) |
| Midwest | 2002 | 0.793 (0.708-0.878) |
| Midwest | 2003 | 0.894 (0.806-0.983) |
| Midwest | 2004 | 0.946 (0.856-1.037) |
| Midwest | 2005 | 1.153 (1.054-1.252) |
| Midwest | 2006 | 1.196 (1.096-1.296) |
| Midwest | 2007 | 1.189 (1.09-1.289) |
| Midwest | 2008 | 1.242 (1.14-1.343) |
| Midwest | 2009 | 1.308 (1.205-1.411) |
| Midwest | 2010 | 1.311 (1.207-1.415) |
| Midwest | 2011 | 1.486 (1.377-1.595) |
| Midwest | 2012 | 1.564 (1.453-1.676) |
| Midwest | 2013 | 1.702 (1.586-1.818) |
| Midwest | 2014 | 1.745 (1.631-1.86) |
| Midwest | 2015 | 1.929 (1.808-2.05) |
| Midwest | 2016 | 2.265 (2.134-2.397) |
| Midwest | 2017 | 2.52 (2.384-2.656) |
| Midwest | 2018 | 2.643 (2.505-2.781) |
| Midwest | 2019 | 2.794 (2.653-2.936) |
| Midwest | 2020 | 4.582 (4.401-4.762) |
| Midwest | 2021 | 5.655 (5.452 - 5.858) |
| Midwest | 2022 | 4.61 (4.428 - 4.793) |
| **South** |  |  |
| South | 1999 | 0.368 (0.32-0.417) |
| South | 2000 | 0.457 (0.404-0.509) |
| South | 2001 | 0.445 (0.395-0.495) |
| South | 2002 | 0.616 (0.555-0.676) |
| South | 2003 | 0.607 (0.55-0.665) |
| South | 2004 | 0.688 (0.626-0.75) |
| South | 2005 | 0.814 (0.749-0.88) |
| South | 2006 | 0.805 (0.739-0.87) |
| South | 2007 | 0.871 (0.805-0.937) |
| South | 2008 | 0.915 (0.846-0.984) |
| South | 2009 | 1.058 (0.986-1.131) |
| South | 2010 | 0.995 (0.925-1.066) |
| South | 2011 | 1.04 (0.969-1.111) |
| South | 2012 | 1.028 (0.959-1.097) |
| South | 2013 | 1.118 (1.047-1.189) |
| South | 2014 | 1.131 (1.06-1.202) |
| South | 2015 | 1.156 (1.086-1.226) |
| South | 2016 | 1.404 (1.327-1.482) |
| South | 2017 | 1.577 (1.495-1.658) |
| South | 2018 | 1.635 (1.552-1.717) |
| South | 2019 | 1.81 (1.723-1.896) |
| South | 2020 | 3.12 (3.008-3.231) |
| South | 2021 | 4.236 (4.105 - 4.367) |
| Sout | 2022 | 2.988 (2.881 - 3.095) |
| **West** |  |  |
| West | 1999 | 0.371 (0.307-0.435) |
| West | 2000 | 0.436 (0.368-0.503) |
| West | 2001 | 0.5 (0.429-0.57) |
| West | 2002 | 0.671 (0.587-0.755) |
| West | 2003 | 0.761 (0.675-0.846) |
| West | 2004 | 0.87 (0.78-0.961) |
| West | 2005 | 0.924 (0.833-1.015) |
| West | 2006 | 1.159 (1.057-1.262) |
| West | 2007 | 1.174 (1.072-1.276) |
| West | 2008 | 1.274 (1.17-1.377) |
| West | 2009 | 1.355 (1.249-1.461) |
| West | 2010 | 1.583 (1.47-1.697) |
| West | 2011 | 1.67 (1.555-1.784) |
| West | 2012 | 1.83 (1.711-1.95) |
| West | 2013 | 1.887 (1.766-2.007) |
| West | 2014 | 1.973 (1.852-2.093) |
| West | 2015 | 2.243 (2.117-2.369) |
| West | 2016 | 2.431 (2.3-2.562) |
| West | 2017 | 2.92 (2.779-3.062) |
| West | 2018 | 2.807 (2.669-2.944) |
| West | 2019 | 2.941 (2.801-3.081) |
| West | 2020 | 5.252 (5.066-5.439) |
| West | 2021 | 6.923 (6.708 - 7.138) |
| West | 2022 | 5.204 (5.021 - 5.388) |

**Supplementary Figure 1: Type 2 Diabetes Mellitus and Obesity-Related Annual Percentage Change (APC) in the United States from 1999 to 2022 stratified by (A) Region (B) Sex (C) Race and (D) Urbanization**

**A)
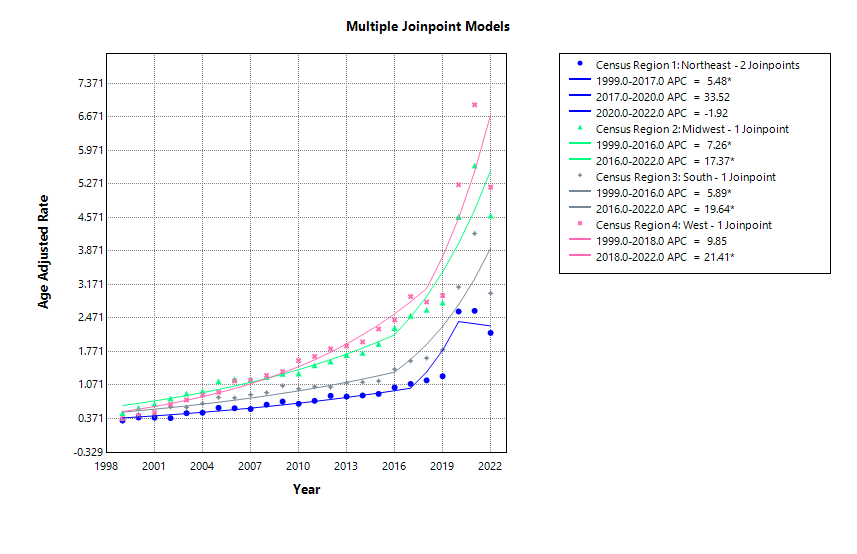
**

**B)
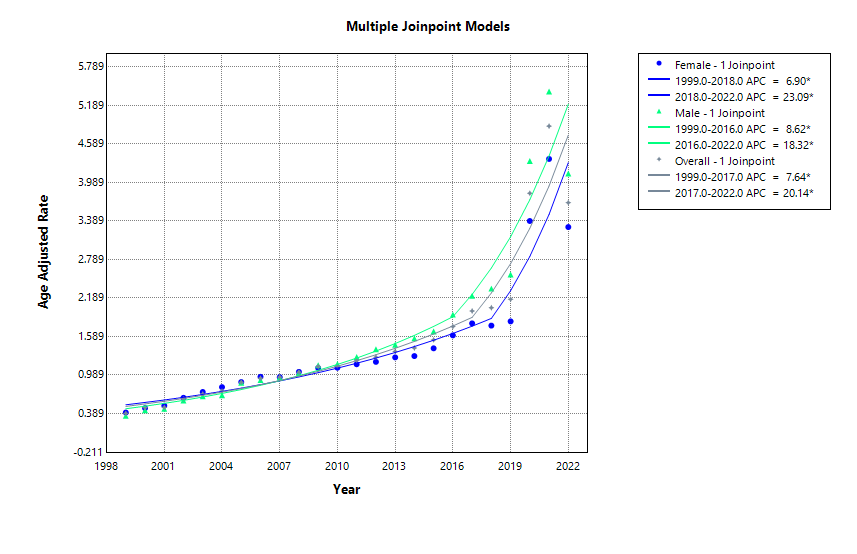
**

**C)
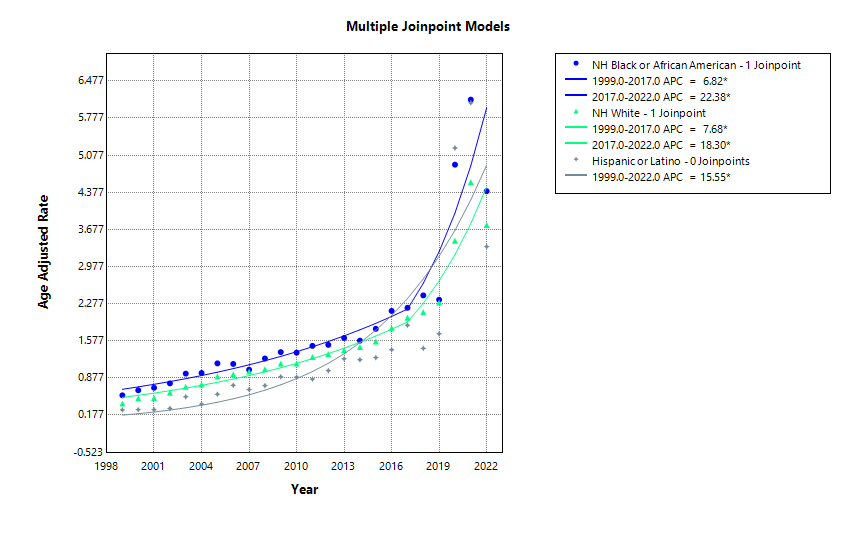
**

**D)
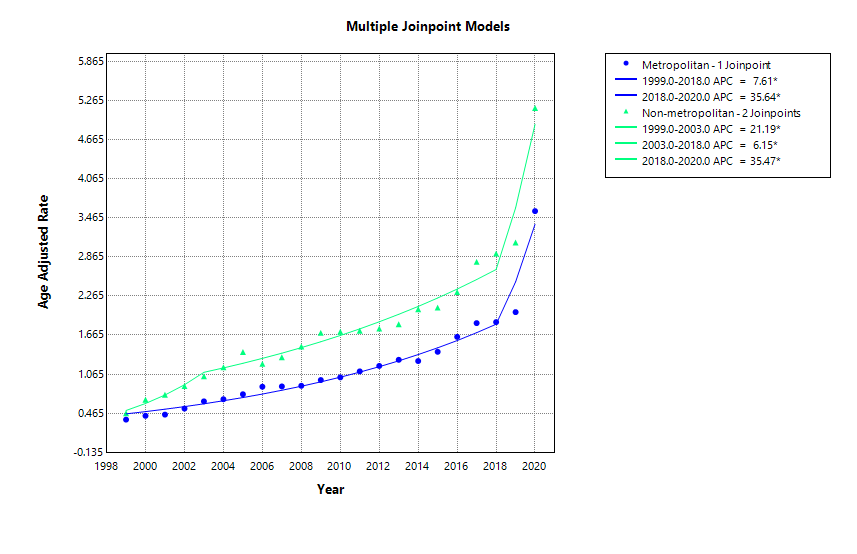
**

**Supplemental Table 7: Type 2 Diabetes mellitus and Obesity Related Age-Adjusted Mortality Rates per 100,000 in United States stratified by Urban-Rural Classification, 1999-2020**

|  | **Age-Adjusted Rate (95% CI)** | |
| --- | --- | --- |
| **Year** | **Urban** | **Rural** |
| 1999 | 0.366 (0.334-0.397) | 0.464 (0.391-0.537) |
| 2000 | 0.425 (0.391-0.458) | 0.673 (0.583-0.762) |
| 2001 | 0.443 (0.410-0.477) | 0.749 (0.654-0.843) |
| 2002 | 0.536 (0.500-0.573) | 0.882 (0.781-0.983) |
| 2003 | 0.647 (0.606-0.688) | 1.034 (0.924-1.144) |
| 2004 | 0.679 (0.638-0.720) | 1.172 (1.056-1.288) |
| 2005 | 0.756 (0.714-0.797) | 1.406 (1.280-1.531) |
| 2006 | 0.872 (0.826-0.917) | 1.223 (1.107-1.338) |
| 2007 | 0.876 (0.832-0.921) | 1.325 (1.205-1.444) |
| 2008 | 0.886 (0.842-0.930) | 1.491 (1.365-1.618) |
| 2009 | 0.974 (0.928-1.020) | 1.700 (1.565-1.835) |
| 2010 | 1.016 (0.968-1.063) | 1.716 (1.580-1.852) |
| 2011 | 1.108 (1.059-1.156) | 1.733 (1.598-1.867) |
| 2012 | 1.192 (1.142-1.242) | 1.765 (1.630-1.899) |
| 2013 | 1.286 (1.234-1.337) | 1.833 (1.695-1.971) |
| 2014 | 1.267 (1.217-1.317) | 2.064 (1.918-2.211) |
| 2015 | 1.409 (1.357-1.461) | 2.088 (1.941-2.235) |
| 2016 | 1.638 (1.582-1.695) | 2.327 (2.173-2.480) |
| 2017 | 1.850 (1.791-1.909) | 2.792 (2.623-2.962) |
| 2018 | 1.865 (1.807-1.924) | 2.920 (2.749-3.091) |
| 2019 | 2.019 (1.958-2.079) | 3.090 (2.915-3.265) |
| 2020 | 3.572 (3.492-3.652) | 5.157 (4.928-5.386) |

Data for urbanization is not available for 2021-2022

**Supplemental Table 8: Deaths due to T2D as primary or contributing cause of death**

| **Year** | **Deaths** | **Age-Adjusted Rate (95% CI)** |
| --- | --- | --- |
| 1999 | 37991 | 21.5(21.3-21.8) |
| 2000 | 41853 | 23.5(23.2-23.7) |
| 2001 | 45383 | 25(24.8-25.2) |
| 2002 | 49162 | 26.7(26.4-26.9) |
| 2003 | 51633 | 27.6(27.3-27.8) |
| 2004 | 53417 | 28.1(27.9-28.3) |
| 2005 | 57678 | 29.8(29.5-30) |
| 2006 | 58663 | 29.7(29.4-29.9) |
| 2007 | 60591 | 30.1(29.8-30.3) |
| 2008 | 63130 | 30.6(30.4-30.9) |
| 2009 | 63807 | 30.4(30.1-30.6) |
| 2010 | 65319 | 30.6(30.3-30.8) |
| 2011 | 65791 | 30(29.8-30.2) |
| 2012 | 67399 | 29.9(29.7-30.1) |
| 2013 | 70206 | 30.4(30.2-30.6) |
| 2014 | 70584 | 29.9(29.7-30.1) |
| 2015 | 77339 | 31.9(31.7-32.2) |
| 2016 | 89210 | 36.1(35.8-36.3) |
| 2017 | 99374 | 39.2(39-39.5) |
| 2018 | 105303 | 40.6(40.3-40.8) |
| 2019 | 111411 | 42(41.8-42.3) |
| 2020 | 155630 | 57.7(57.4-58) |
| 2021 | 165420 | 62.7(62.4-63 |
| 2022 | 156826 | 56.9(56.6-57.2 |
